# Extra-Chromosomal DNA Sequencing Reveals Episomal Prophages Capable of Impacting Virulence Factor Expression in *Staphylococcus aureus*

**DOI:** 10.3389/fmicb.2018.01406

**Published:** 2018-07-02

**Authors:** Douglas R. Deutsch, Bryan Utter, Kathleen J. Verratti, Heike Sichtig, Luke J. Tallon, Vincent A. Fischetti

**Affiliations:** ^1^Laboratory of Bacterial Pathogenesis and Immunology, The Rockefeller University, New York, NY, United States; ^2^Applied Physics Laboratory, National Security Systems Biology Center, Johns Hopkins University, Laurel, MD, United States; ^3^Center for Devices and Radiological Health, Office of In Vitro Diagnostics, U.S. Food and Drug Administration, Silver Spring, MD, United States; ^4^Genomics Resource Center, Institute for Genome Sciences, University of Maryland School of Medicine, Baltimore, MD, United States

**Keywords:** bacteriophages, extra-chromosomal DNA, episomal, plasmidial, active lysogeny, virulence factor

## Abstract

*Staphylococcus aureus* is a major human pathogen with well-characterized bacteriophage contributions to its virulence potential. Recently, we identified plasmidial and episomal prophages in *S. aureus* strains using an extra-chromosomal DNA (exDNA) isolation and sequencing approach, uncovering the plasmidial phage ϕBU01, which was found to encode important virulence determinants. Here, we expanded our extra-chromosomal sequencing of *S. aureus*, selecting 15 diverse clinical isolates with known chromosomal sequences for exDNA isolation and next-generation sequencing. We uncovered the presence of additional episomal prophages in 5 of 15 samples, but did not identify any plasmidial prophages. exDNA isolation was found to enrich for circular prophage elements, and qPCR characterization of the strains revealed that such prophage enrichment is detectable only in exDNA samples and would likely be missed in whole-genome DNA preparations (e.g., detection of episomal prophages did not correlate with higher prophage excision rates nor higher excised prophage copy numbers in qPCR experiments using whole-genome DNA). In *S. aureus* MSSA476, we found that enrichment and excision of the prophage ϕSa4ms into the cytoplasm was temporal and that episomal prophage localization did not appear to be a precursor to lytic cycle replication, suggesting ϕSa4ms excision into the cytoplasm may be part of a novel lysogenic switch. For example, we show that ϕSa4ms excision alters the promoter and transcription of *htrA_2_*, encoding a stress-response serine protease, and that alternative promotion of *htrA_2_* confers increased heat-stress survival in *S. aureus* COL. Overall, exDNA isolation and focused sequencing may offer a more complete genomic picture for bacterial pathogens, offering insights into important chromosomal dynamics likely missed with whole-genome DNA-based approaches.

## Introduction

*Staphylococcus aureus* is a Gram-positive human pathogen with an arsenal of virulence factors allowing its successful colonization and infection of hosts (Thammavongsa et al., 2015; Thomer et al., 2016). Prophages and their encoded factors, in particular, play a significant role in the virulence potential and adaptability of the pathogen by means of lysogenic (positive) conversion, or the modulation of bacterial phenotypes by phage-encoded genes. *S. aureus* Newman, for example, displays severely reduced virulence in a mouse model when cured of its four prophages (Bae et al., 2006). Some prophages in particular have increased roles in the pathogenicity of strains, as they can positively convert cells with additional virulence determinants. The β-hemolysin (*hlb*)-converting phages of *S. aureus* carry genes for the well characterized toxins staphylokinase (SAK) and enterotoxin A (SEA) (Coleman et al., 1989), as well as the more recently discovered chemotaxis inhibitory protein of *S. aureus* (CHIPS) and staphylococcal complement inhibitor (SCIN), which allow bacterial evasion of the innate immune system (van Wamel et al., 2006). Other phages carry toxins such as the pore-forming Panton-Valentine leukotoxin (PVL) or exfoliative toxin A (ETA), the causative agent of staphylococcal scalded skin syndrome (SSSS) (Kaneko et al., 1998; Yamaguchi et al., 2000). Lysogenic conversion can also result from the disruption of host-encoded virulence factors through prophage genome integration (negative conversion). The *hlb*-converting phages integrate within and disrupt the β-hemolysin encoding gene of *S. aureus* (negatively converting cells for β-hemolysis), and similarly, lipase (*geh*)-converting phages, such as ϕL54a, negatively convert cells for lipase activity by integration within the lipase (*geh*) gene (Lee and Iandolo, 1986). The disruption of these genes has been shown to affect the colonization and virulence potential of *S. aureus* (Hu et al., 2012; Katayama et al., 2013), and while *hlb*-converting phages indeed encode alternative virulence determinants, *geh*-converting phages do not encode known virulence factors, with the benefits of their carriage unclear.

While positive and negative phage-conversion are well studied events, recent reports have also focused on the excision/integration dynamics and atypical localizations of temperate phages in *S. aureus* (Goerke et al., 2004, 2006; Utter et al., 2014). In *S. aureus* isolates from cystic fibrosis and bacteremic patients, genomic alterations driven by *hlb*-converting phages were found to occur, whereas *S. aureus* isolates from the noses of healthy individuals showed minimal changes. Specifically, in isolates from diseased patients, *hlb*-converting phages were found integrated at atypical chromosomal loci, resulting in Hlb^+^/sak^+^ phenotypes (cells with an intact *hlb* gene, but positively converted by prophages encoding *sak*). Rarely, in some strains, phages appeared to undergo duplication and dual integration, generating populations with a Hlb^-^/sak^2^ phenotype (cells with one prophage integrated in *hlb* and a duplicated prophage integrated in another chromosomal location, with both phages encoding *sak*) (Goerke et al., 2006). However, such phage mobilization occurred to a significantly lesser degree in nasal isolates from healthy individuals (colonizing but not infectious isolates), indicating selective pressure for Hlb-producing strains in the transition to invasive infection, with atypical prophage integration as a mechanism to allow for dual Hlb and SAK production. Previous work in our laboratory uncovered the plasmidial prophage ϕBU01, with a DNA sequence containing high homology to known *hlb*-converting phages. ϕBU01 did not appear to integrate within the *S. aureus* chromosome (Utter et al., 2014), suggesting that in addition to atypical chromosomal integration, maintenance of prophages in the extra-chromosomal compartment can result in an Hlb^+^/sak^+^ phenotype (Deutsch et al., 2016).

Excision/integration dynamics of temperate phage (or phage-like elements) can also have important roles in other bacterial pathogens. *Streptococcus pyogenes* strain SF370 contains the episomal phage-like chromosomal island, SpyCIM1 (formerly termed ϕ370.4; Beres and Musser, 2007), which integrates within the cell’s DNA mismatch repair operon, disrupting transcription of the *mutS-mutL* genes and consequently increasing the mutation rate of the cell approximately 200-fold (Scott et al., 2008; Hendrickson et al., 2015). The phage-like element was found to be excised at low cellular densities allowing faithful genome replication, but would integrate and increase the cell’s mutation rate at higher cell densities (Scott et al., 2008)—conditions where mutation might be beneficial (i.e., low nutrient availability). SpyCIM1’s temporal dynamics suggest that *S. pyogenes* is using the phage-like element for its own benefit as a molecular switch at the DNA-level. Other species (*Listeria monocytogenes, Bacillus subtilis*) have been found to employ similar strategies with their phages, allowing control of activities such as phagosomal escape (Rabinovich et al., 2012) and sporulation (Kunkel et al., 1990; Takemaru et al., 1995), respectively. Feiner et al. (2015) reviewed these and other similar temperate phage–bacteria interactions, where prophage excision/integration generates genomic switches with significant impacts on the host, terming it “active lysogeny.”

Previously, we screened clinical isolates of *S. aureus* for the presence of rare, cytoplasmically localized prophages using extra-chromosomal DNA (exDNA) enrichment and next-generation sequencing (NGS). In this earlier report (Utter et al., 2014), we identified and sequenced the plasmidial prophage ϕBU01 from the vancomycin-intermediate *S. aureus* (VISA) NRS19, and found that the phage encoded multiple virulence determinants; we also uncovered an episomal prophage from VISA NRS26 in the same study. Enrichment and screening of the cytoplasmic compartment revealed that extra-chromosomally localized prophages were fairly widespread in *S. aureus*, and that such “hidden” elements may alter the virulence potential of a strain. For our current study, we expanded our screen for extra-chromosomal prophages in *S. aureus*, employing similar NGS methods to screen-by-sequencing an additional 15 diverse, clinical isolates. Unlike our previous work, however, the strains selected for this study had previously sequenced and assembled chromosomes, allowing the classification of prophages uncovered by our method as either episomal (found both integrated and extra-chromosomal in a population) or plasmidial (solely extra-chromosomal). Here, we found extra-chromosomal prophages present in 5 of 15 strains, but surprisingly, these strains contained only episomal and not plasmidial prophages. Furthermore, we demonstrate that the episomal phages uncovered by our approach are circular DNA elements, and reveal that enrichment and detection of such phages would not occur using conventional whole-genome DNA preparations; PCR-based measurements of excised phage copy numbers and excision rates from whole-genome DNA preparations did not correlate with enrichment in our screening. In addition, we find cytoplasmic localization of ϕSa4ms, from the *S. aureus* strain MSSA476, to be growth-phase dependent and that the prophage does not appear to replicate after excision, suggesting it exists in a state of “active lysogeny.” Lastly, we show that excision of ϕSa4ms alters the promoter sequence and transcription of the stress-response serine protease-encoding *htrA_2_*, with promoter alterations affecting heat-stress survival in *S. aureus* COL. Thus, our extra-chromosomal enrichment and sequencing approach allows the detection of these “active” prophages and other episomal or plasmidial DNA elements, offering greater insights into the virulence potential and genome dynamics of an organism.

## Materials and Methods

### Bacterial Strains and Growth Conditions

Strains used in this study are listed in **Supplementary Table S1**. *S. aureus* strains were established from overnight (O/N) cultures grown in Bacto brain heart infusion (BHI), and back-diluted 1:100 into 50 mL BHI without shaking at 37°C unless otherwise noted. For preparation of exDNA, strains were grown as described to an OD_600_ of 0.6–0.8. Cultures were centrifuged at 4000 rpm for 10 min at 4°C and used immediately or frozen O/N at -20°C. For qPCR studies, strains were back-diluted from O/N cultures and grown to specified optical densities. For P*htrA_2_*-GFP reporter studies, O/N cultures were back-diluted 1:100 and grown at 200 rpm at 37°C to an OD_600_ = 0.2. Concentrations for antibiotics used are as follows, for *Escherichia coli*: ampicillin (100 μg/mL); for *S. aureus*: erythromycin (5 μg/mL), spectinomycin (50 μg/mL). For heat shock studies, plates were incubated at 44°C.

### Whole-Genome and exDNA Isolation and Manipulation

Whole-genome DNA isolations (gDNA) were performed using the QIAGEN DNeasy Blood and Tissue Kit, which included an added manufacturer-detailed pretreatment step for Gram-positive bacteria. Enzymatic lysis buffer was composed of lysostaphin (100 μg/mL) in 1× phosphate-buffered saline (PBS). exDNA isolation was carried out as previously described (Utter et al., 2014). exDNA samples were visualized on 0.7% agarose 0.5× TAE gels stained with SYBR Safe DNA Gel Stain. Electrophoresis was carried out at 50 V for 1 h in 0.5× TAE, and visualized with UV transillumination. Prior to DNA sequencing, exDNA samples were concentrated as needed using Microcon DNA Fast Flow (EMD Millipore) centrifugal filters, following manufacturer’s directions.

### DNA Sequencing of Extra-Chromosomal *S. aureus* Samples

DNA sample concentrations were measured using the Thermo Fisher Scientific Qubit Fluorometer High Sensitivity DNA kit. Size and quality of DNA was measured using the Agilent Technologies Bioanalyzer High Sensitivity DNA assay. DNA libraries were constructed as per manufacturer’s instructions starting with 1 ng of DNA per sample, using the Illumina Nextera XT DNA Library Preparation Kit and the Nextera XT Index Kit. The completed DNA libraries were quality checked using the Agilent Technologies High Sensitivity DNA assay. Shotgun sequencing of the DNA libraries was performed using the Illumina MiSeq Reagent kit V2 (500 cycle) on the Illumina MiSeq sequencer. Prior to sequencing, libraries were normalized and pooled together to make a multiplexed pooled DNA library at 2 nM concentration. The 2 × 250 paired-end sequencing run generated FASTQ files to allow for off-instrument analysis. Raw extra-chromosomal DNA sequencing reads associated with this study are accessible via NCBI BioProject PRJNA475753.

### Bioinformatic Sequence Analysis of exDNA Samples

Bioinformatic analysis was performed using CLC Genomics Workbench software unless otherwise described. Extra-chromosomal reads were mapped to respective chromosomal sequences (**Supplementary Table S1**), and unmapped reads were saved as a separate file, and *de novo* assembled for identification of possible plasmidial elements. Read mappings were visually examined for regions of increased read depth, corresponding to DNA element enrichment in exDNA samples. Read mappings were then subjected to coverage analysis using CLC Genomics Workbench software to identify regions (minimum length 500 bp) with significantly higher (*P* < 0.05) coverage distributed throughout integrated prophage genome locations. Prophage and other mobile DNA element regions with high coverage were noted for each sequenced sample.

### qPCR Analysis of *S. aureus* Strains

For qPCR experiments, strains were grown as described for exDNA sequenced samples, or to other desired optical densities. Cultures were divided, with one portion of the culture subjected to the QIAGEN DNeasy Blood and Tissue Kit for gDNA isolation, and the rest undergoing exDNA isolation. Whole-genome DNA samples were used to determine excision rates and phage or plasmid (pSAS1) copy numbers per cell of the bacterial population, while exDNA samples were used only for phage and plasmid copy number measurements. Primer pairs and probe sequences for each target are listed in **Supplementary Table S2**. All primers and probes were designed and purchased from Integrated DNA Technologies (Coralville, IA, United States). Amplification was carried out using the TaqMan Gene Expression Master Mix (Thermo Fisher Scientific) and the Life Technologies QuantStudio 12K-Flex Instrument following manufacturer’s cycling protocol. A standard curve for each primer-probe set was set up for each experimental run, with amplification efficiencies and linear regression analyzed using QuantStudio Software. All primer-probe sets had efficiencies 90–110% and *R*^2^ > 0.98. Excision rates, excised prophage copy numbers, and plasmid copy numbers were calculated by normalizing the targets (*attB, attP*, pSAS1, respectively) to *gyrA*. All targets were measured in biological triplicate and technical duplicates. Graphical and statistical analysis was performed using GraphPad Prism, with significance testing done using two-tailed Student’s *t*-tests. Bar graphs are presented as mean ± standard error of the mean (SEM).

### Linear DNase and Restriction Endonuclease Treatment of exDNA Samples and End-Point PCR of DNA Targets

Prior to linear DNase and/or treatment with restriction endonuclease, exDNA samples were treated with PreCR Repair Mix (NEB) to repair nicked DNA. PreCR-treated samples were then either (1) treated with the ϕSa4ms-specific restriction endonucleases *Psh*AI and *Psp*XI (NEB) following manufacturer’s protocol, (2) treated with Plasmid-Safe-ATP-Dependent DNase (Epicentre), (3) treated with *Psh*AI and *Psp*XI, then the Plasmid-Safe-ATP-Dependent DNase, or (4) left solely PreCR-treated. Plasmid-Safe treated samples were treated over the course of 16 h at 37°C, where 2 μL ATP solution and 2 μL DNase were added at 2 and 4 h time points. Samples were then heated to inactivate enzymes prior to PCR. End-point PCR was carried out using target-specific primers (**Supplementary Table S2**) and the KAPA2G Robust Hotstart Polymerase (KAPA Biosystems) with the following cycling protocol: (1) initial denaturation 95°C, 3 min; (2) denaturation 95°C, 15 s; (3) annealing 60°C, 15 s; (4) extension 72°C, 15s; (5) repeat steps 2–4 thirty three times. Samples were normalized prior to loading individual PCRs to account for dilutions from original sample concentration due to enzyme treatments. PCR products were visualized using 1% agarose 0.5× TAE gels stained with SYBR Safe DNA Gel Stain. Electrophoresis was carried out at 100 V for 25 min in 0.5× TAE, and visualized with UV transillumination. Images were captured using Alpha Imager HP software.

### Construction and Testing of P*htrA_2_*-GFP Reporter System and *htrA_2_*-Complemented Knockouts

For construction of a P*htrA_2_*-GFP reporter system, 250 bp upstream of the *htrA_2_* gene plus 30 bp of *htrA_2_* were amplified from a MSSA476 gDNA template using Phusion High-Fidelity DNA Polymerase with two primer sets (1) Pint_upstm/P_dwnstm or (2) Pex_upstm/P_dwnstm (**Supplementary Table S2**) to amplify two versions of the *htrA_2_* promoter and 5′ gene region (P*int* or P*ex*). PCR products were gel purified, cut with restriction enzymes *Kpn*I and *Bam*HI (NEB), and further purified. pCN56 vector (Charpentier et al., 2004) was purified using the QIAprep Spin Miniprep Kit (QIAGEN), digested with *Kpn*I and *Bam*HI, dephosphorylated with Antarctic Phosphatase (NEB), and gel purified before ligation using T4 ligase (NEB) with the purified PCR products. Next, 2 μL of the ligation mixture was introduced into One Shot TOP10 Chemically Competent *E. coli* (Thermo Fisher Scientific) and the resulting colonies screened by PCR and Sanger sequencing (GENEWIZ; South Plainfield, NJ, United States), using primers Pint_upstm_seq/P_dwnstm_seq or Pex_upstm_seq/P_dwnstm_seq for the desired vector insert (**Supplementary Table S2**). *E. coli* cultures harboring pCN56 with P*int* or P*ex* inserts were grown and miniprepped, and the purified vectors electroporated into electrocompetent *S. aureus* RN4220 (Kreiswirth et al., 1983) using a Bio-Rad Gene Pulser with the following settings: 2.5 kV, 25 μF, 100 Ω. Empty pCN56 vector was also introduced into RN4220. Vectors from RN4220 colonies were then transduced, using ϕNM4γ4 (Heler et al., 2015), into *S. aureus* COL *htrA_2_* (Rigoulay et al., 2005) following an established protocol (Olson, 2016). Colonies were screened via PCR and inserts Sanger sequenced to ensure the correct DNA sequence.

To examine GFP fluorescence in each strain, O/N cultures of constructs were back-diluted 1:100 in 15 mL BHI supplemented with 5 μg/mL erythromycin and 50 μg/mL spectinomycin and grown at 37°C, 200 rpm to an OD_600_ = 0.2. Cultures were then grown for 3 h at 37°C or 44°C with shaking. After 3 h growth, 1 mL of culture was removed, an OD_600_ value measured, and 200 μL of culture was pipetted into a quartz 96-well plate to measure RFUs on a Molecular Devices SpectraMax M5 instrument (485 nm excitation, 515 nm emission). Fluorescence of pCN56 (empty vector) cultures were subtracted as background, and OD_600_ normalized RFUs for each sample were measured. Comparison of construct-GFP fluorescence was calculated as a ratio of P*ex*/P*int*. Graphical and statistical analysis was performed using Prism GraphPad. Bar graphs are presented as mean ± SEM, with significance indicated above each bar. Significance testing was performed using two-tailed ratio paired *t*-tests. The experiment was performed at least in triplicate.

For construction of *htrA_2_*–complement vectors, the *htrA_2_* gene and 250 bp upstream of the gene were amplified using the primer set (1) Pint_upstm/full_htrA2_dwnstm or (2) Pex_upstm/full_htrA2_dwnstm (**Supplementary Table S2**) with Q5 DNA Polymerase (NEB) to generate two DNA fragments containing the full length *htrA_2_* sequence with different 250 bp promoter sequences. PCR products were treated as described above, before being ligated into the pCN35 vector (Charpentier et al., 2004). The ligated vectors and pCN35 empty vector were processed as above, introduced into electrocompetent *E. coli* DC10B, shuttled into RN4220, transduced into *S. aureus* COL *htrA_2_*, then screened and sequenced as previously described using primers full_htrA2_seq1, full_htrA2_seq2, full_htrA3_seq3, full_htrA2_dwnstm, Pint_upstm_seq, and Pex_upstm_seq (**Supplementary Table S2**).

To test viability and heat-stress survival of complemented and mutant (empty vector-containing) *S. aureus* COL *htrA_2_* strains, constructs were spotted in a log-dilution series onto BHI–spectinomycin–erythromycin agar plates and incubated at 37 and 44°C for 24 h following a protocol for determining heat sensitivity from (Rigoulay et al., 2005). Plates were examined visually for survival and photographed the following day using a Cell Biosciences AlphaImager HP instrument using AlphaImager HP software. The experiment was performed in triplicate.

## Results

### Extra-Chromosomal DNA Isolation and Sequencing Reveals the Presence of Episomal Prophage Elements Within *S. aureus* Clinical Isolates

**FIGURE 1:**
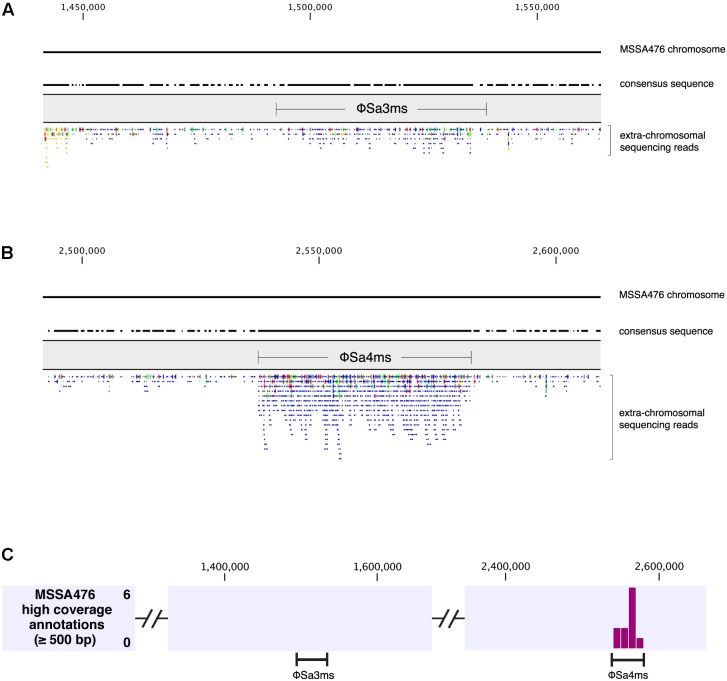
Read mapping and coverage analysis of MSSA476 extra-chromosomal DNA sequencing. **(A,B)** Read mappings of sequencing reads for MSSA476 exDNA. ϕSa4ms **(B)** has greater read coverage compared to ϕSa3ms **(A)** in exDNA sequencing. Selected portions of the read mapping surrounding prophage integration sites for ϕSa3ms **(A)** and ϕSa4ms **(B)** are shown. Chromosomal positions are labeled at top, and consensus regions are shown below for MSSA476 chromosome sequence and MSSA476 exDNA reads. Locations of ϕSa3ms **(A)** and ϕSa4ms **(B)** integrated prophage genomes are shown. Extra-chromosomal reads are shown below for each. **(C)** Coverage analysis histogram from MSSA476 extra-chromosomal DNA sequencing read mapping. Portions of coverage analysis histogram surrounding prophage regions shown for clarity. Chromosomal positions labeled at top of image, and approximate positions of ϕSa3ms and ϕSa4ms genomes shown below. Histogram of higher read-coverage regions on the MSSA476 chromosome contains 11 significantly higher coverage regions within the ϕSa4ms prophage genome location, indicating prophage enrichment in exDNA sequencing. Read-mapping across ϕSa3ms prophage genome location does not contain any regions of significantly higher coverage, indicating no prophage enrichment by exDNA isolation and sequencing.

For this study, we expanded our previously developed exDNA enrichment and sequencing approach to screen *S. aureus* clinical isolates for prophage elements present in the extra-chromosomal compartment of the cell (Utter et al., 2014). We selected 15 clinical isolates sourced from different worldwide geographic regions, containing a diverse array of antibiotic resistances and virulence factors. In addition, these strains had fully sequenced chromosomes, facilitating the classification of any detected prophages as episomal or plasmidial. We enriched and prepared exDNA of these strains as described in Section “Materials and Methods,” and performed paired-end sequencing using the Illumina MiSeq sequencer. exDNA samples were analyzed by first mapping sequencing reads to their corresponding chromosomal sequences, followed by *de novo* assembly of unmapped reads. Visual examination of chromosomal read mappings revealed areas of increased sequencing coverage, especially over some prophage regions (**Figures 1A,B**). Coverage analysis transformed visual read-mapping data into histograms and highlighted ≥500 bp regions of the chromosome where read depth was significantly increased (*P* < 0.05). Such regions of higher coverage within integrated prophage genomes indicated the presence of episomal elements (and/or potentially active prophages) enriched in sequencing due to specifically targeting and isolating DNA from the extra-chromosomal compartment of the cell (**Figure 1C**). Read mappings and a coverage analysis histogram are shown for strain MSSA476 as a representative example (**Figure 1**).

For each strain, we documented whether any prophage elements were enriched in sequencing due to exDNA isolation and classified the strains as: (1) containing enriched prophage elements, (2) not containing enriched prophage elements, or (3) containing unclear prophage element enrichment, when partial but not complete prophage regions had increased coverage detected (**Table 1**). Surprisingly, *de novo* assembly of unmapped reads did not reveal any new prophage elements, suggesting no plasmidial prophages were present in the sequenced strains, and that all prophages uncovered in the extra-chromosomal compartment by our approach were episomal elements. Importantly, episomal prophages were identified in one-third (5 of 15) of the staphylococcal strains analyzed. These included: (1) MSSA476, harboring ϕSa4ms (Sa4-like integrase), (2) NRS143 containing a *geh*-converting prophage (Sa6-like integrase), (3) BK2529 harboring a prophage with a Sa7-like integrase, as well as two strains each with three episomal prophages. One of these strains, HPV107, contained episomal prophages with Sa2- and Sa3-like integrases, and in addition, one prophage with an unclear integrase type, however, its sequence was homologous to the integrase of a prophage from *S. aureus* SA268 (Qu et al., 2014). The other strain, NRS22, contained episomal prophages with Sa2-, Sa5-, and Sa7-like integrase sequences. Interestingly, when integrated into the chromosome, the Sa2-like prophage of HPV107 disrupts a 6-phospho-β-galactosidase encoding gene, which to our knowledge is a novel integration site for a *S. aureus* prophage. The other episomal phages we uncovered had chromosomal integration sites that have been described previously (Bae et al., 2006; Goerke et al., 2006, 2009). In addition to these episomal prophage elements, we also uncovered an enriched integrative and conjugative element (ICE*6013*-like) typically located within a predicted membrane protein encoding locus in NRS271, however, its existence as an extra-chromosomal element has been previously reported (**Table 1**) (Smyth and Robinson, 2009; Sansevere et al., 2017). Incidentally, known plasmids, such as pSAS1 in MSSA476, were *de novo* assembled by using unmapped reads, and while they are not the focus of this current study, they do point to the sensitivity of this exDNA isolation and sequencing method.

**Table 1:** Detection of enriched prophages from extra-chromosomal DNA sequencing.

Strain	Resistances	#Prophage regions	Notes
**Enriched prophage elements detected**			
BK2529	MRSA	3	• One enriched prophage: Sa7-like integrase, intergenic between *rpmF* and *isdB*
HPV107	MRSA	3	Three enriched prophages: • Sa2-like integrase, intragenic in RL05_04730 (6-phospho-β-galactosidase) • Sa3-like integrase, *hlb*-converting phage (ϕHPV107.1) • Unclear integrase type, intergenic between RL05_02285 (tRNA-Ser) and RL05_01940 (enterotoxin)
MSSA476	MSSA	2	• ΦSa4ms enriched, integrates 30 bp upstream of *htrA_2_* • ΦSa3ms not enriched, *hlb*-converting
NRS22	VISA/MRSA	4	Three enriched prophages: • Sa2-like integrase, intragenic in RK87_02365 (hypothetical protein) • Sa5-like integrase, intragenic in RK87_04825 (radical SAM) • Sa7-like integrase, intergenic between *rpmF* and *isdB*
NRS143	MSSA	2	• One enriched prophage: Sa6-like integrase, *geh*-converting
**Unclear enriched prophage elements**			
NRS2	VISA/MRSA	2	• Unclear enrichment of Sa7-like integrase prophage, intergenic between *rpmF* and *isdB*
NRS153	MSSA	3	• Unclear enrichment of Sa1-like integrase prophage, intergenic between *sufB* and RK79_06750 (transposon-encoded integrase)
NRS387	MRSA	1	• Unclear enrichment of Sa3-like integrase prophage, *hlb*-converting
**No enriched prophage elements detected**			
BAA-42	MRSA	3	
E2125	MRSA	3	
HDE288	MRSA	3	
NRS127	MRSA	5	
NRS156	MSSA	1	
NRS158	MSSA	2	
NRS271	MRSA	2	• Enriched ICE (ICE*6013*-like) within RK77_00405 (membrane protein)

*Relevant resistances (MSSA, MRSA, VISA, VRSA) and total number of predicted chromosomal prophages for each strain are listed*.

### qPCR Characterization of MSSA476 Validates Extra-Chromosomal Sequencing Data, and Also Reveals Episomal ϕSa4ms Is Detectable Only in exDNA Samples

The conditions of our screening-by-sequencing approach did not reveal any non-integrating, plasmidial prophages. However, a number of the clinical strains did contain episomal prophage elements uncovered from the cytoplasmic compartment by specific exDNA isolation (**Table 1**). Thus, we were curious what insights prophage enrichment, as uncovered by exDNA sequencing, revealed about an individual strain. To accomplish this, we chose to focus on the well-characterized *S. aureus* strain MSSA476 (Sumby and Waldor, 2003; Holden et al., 2004) and took a qPCR approach to determine the excision rates and copy numbers of its prophages. MSSA476 contains two prophages, ϕSa3ms and ϕSa4ms. ϕSa3ms is an *hlb*-converting phage, while ϕSa4ms integrates 30 bp upstream of the serine protease-encoding *htrA_2_*. In our sequencing, ϕSa4ms had significantly increased read depth in read-mappings (i.e., was enriched by exDNA isolation), while ϕSa3ms did not (**Figure 1**). Therefore, MSSA476 was ideal to understand the characteristics of both enriched and non-enriched prophages in our screening.

We grew MSSA476 as described in Section “Materials and Methods,” and isolated exDNA and whole-genome DNA (gDNA) from cultures at OD_600_ = 0.35, 0.7, and 1.0, as well as from O/N cultures, and profiled the excision rates and excised copy numbers of the strain’s prophages by qPCR. To determine the excised copy numbers of ϕSa3ms and ϕSa4ms, we targeted prophage attachment sites (*attP*), designing primers to yield a PCR product only when phages were not integrated in the chromosome, and normalized to gene copies of chromosomal DNA gyrase (*gyrA*) for both exDNA and gDNA samples. Excision rates were determined by targeting phage-free bacterial attachment sites on the chromosome (*attB*) and normalized to *gyrA* in gDNA samples. qPCR data for excised copy numbers and excision rates of ϕSa3ms and ϕSa4ms at each growth point are listed in **Table 2**.

**Table 2:** Excised copy numbers and excision rates of MSSA476 prophages at selected optical densities.

Prophage	OD_600_	Excised prophage copy number (copies *attP*/copy *gyrA*)		Excision rate (copies *attB*/copy *gyrA*)
		exDNA	gDNA	
ΦSa3ms	0.35	0.3801 ± 0.050	0.109 ± 0.009	4.64 × 10^-4^ ± 4.52 × 10^-5^
	0.7^∗^	0.3377 ± 0.064	0.095 ± 0.005	4.80 × 10^-4^ ± 1.10 × 10^-5^
	1.0	0.2653 ± 0.021	0.1370 ± 0.013	5.05 × 10^-4^ ± 2.73 × 10^-5^
	O/N	0.2563 ± 0.014	0.1230 ± 0.001	9.58 × 10^-4^ ± 2.14 × 10^-5^
ΦSa4ms	0.35	2.220 ± 0.110	0.0840 ± 0.006	1.51 × 10^-4^ ± 2.47 × 10^-5^
	0.7^∗^	1.257 ± 0.229	0.0647 ± 0.001	9.91 × 10^-5^ ± 3.13 × 10^-6^
	1.0	0.4270 ± 0.030	0.0767 ± 0.008	8.44 × 10^-5^ ± 1.14 × 10^-5^
	O/N	0.2073 ± 0.019	0.0700 ± 0.008	8.75 × 10^-5^ ± 8.15 × 10^-6^

*Asterisk “^∗^” indicates culture density representative of samples from extra-chromosomal DNA sequencing. O/N, overnight culture. Values are displayed as mean ± SEM*.

**FIGURE 2:**
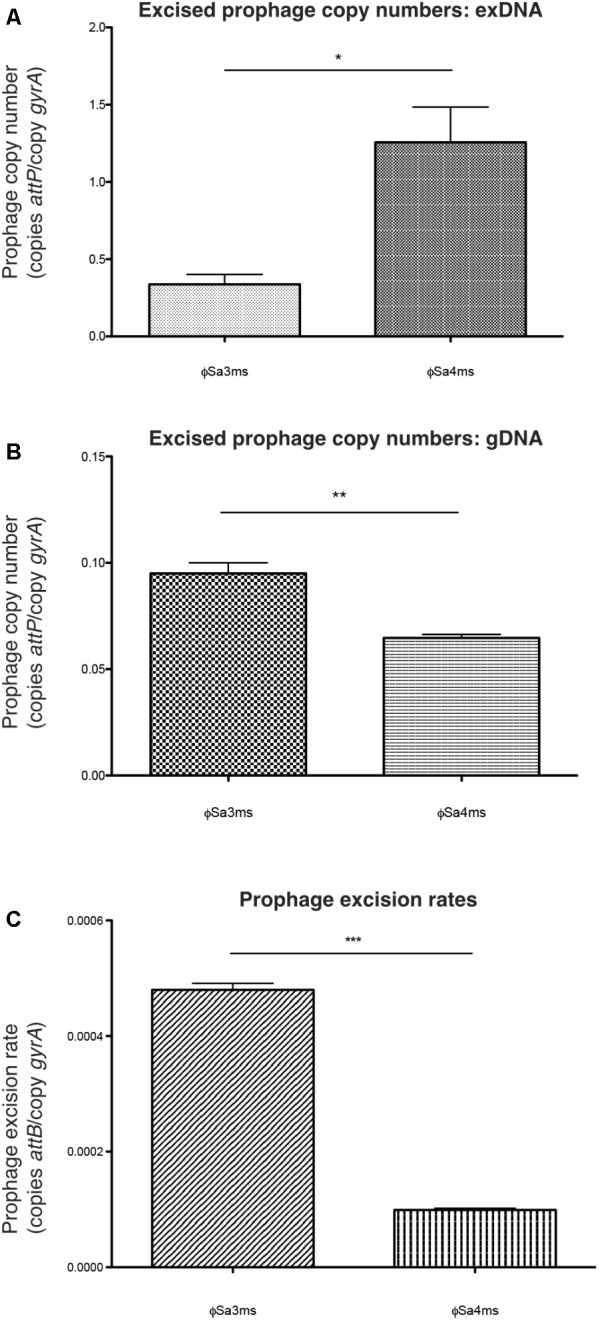
qPCR characterization of MSSA476 excised prophage copy numbers and excision rates at OD_600_ = 0.7. **(A)** Prophage copy numbers (copies *attP*/copy *gyrA*) from MSSA476 extra-chromosomally enriched DNA (exDNA) samples. Prophage copy number is significantly higher for ϕSa4ms than ϕSa3ms, in accordance with sequencing data. **(B)** Prophage copy numbers from MSSA476 whole-genome DNA (gDNA) samples. Copy number of ϕSa3ms is significantly higher than ϕSa4ms. **(C)** Excision rates of ϕSa3ms and ϕSa4ms (copies *attB*/copy *gyrA*). Excision rate of ϕSa3ms is significantly higher than ϕSa4ms. Bars indicate mean ± SEM. ^∗^*P* < 0.05; ^∗∗^*P* < 0.01; and ^∗∗∗^*P* < 0.001.

We first wanted to validate our extra-chromosomal sequencing results, which suggested ϕSa4ms may exist as a stable episome in a portion of the MSSA476 population. Sequencing data should have a direct correlation to excised prophage copy numbers, predicting that ϕSa4ms copy number would be greater than ϕSa3ms in exDNA samples from OD_600_ = 0.7, a growth point representative of when samples were processed for initial exDNA isolation and sequencing. Indeed, the copy number of excised ϕSa4ms (copies *attP*/copy *gyrA*) was significantly higher than ϕSa3ms (*P* = 0.018), supporting our exDNA sequencing data (**Table 2** and **Figure 2A**). Unexpectedly, however, examination of excised prophage copy numbers from gDNA samples revealed that ϕSa3ms excised copy number was significantly higher than that of ϕSa4ms (*P* = 0.0046) (**Table 2** and **Figure 2B**). In addition, excision rate data correlated with the gDNA excised prophage copy numbers; ϕSa3ms had a significantly higher excision rate (copies *attB*/copy *gyrA*) than ϕSa4ms (*P* < 0.0001) (**Table 2** and **Figure 2C**). While the gDNA copy number and excision rate data appeared to be in agreement (i.e., higher excision rates correlate with higher excised prophage copy numbers) they were in contrast to copy number results obtained from qPCR of exDNA samples and from the initial sequencing screening. We therefore performed additional experiments to uncover the basis of these apparent conflicting results before analyzing the remaining qPCR data from OD_600_ = 0.35, 1.0, and O/N cultures.

### Extra-Chromosomal DNA Samples Are Enriched for Circular Prophages

To uncover why ϕSa4ms was enriched by exDNA isolation but not in gDNA preparations, we first compared ϕSa3ms and ϕSa4ms prophage genomes for %GC content, which could alter DNA capture efficiency. However, %GC content did not appear to play a role in enrichment differences as ϕSa3ms and ϕSa4ms contain almost equivalent %GC content at 33.2 and 33.3%, respectively. Part of the extra-chromosomal enrichment protocol involves alkaline-lysis followed by centrifugation. We hypothesized that this step likely enriches for circular forms of prophage that would remain soluble after alkaline-lysis and not pellet during centrifugation (i.e., would be captured in the exDNA preparation). Linear phage DNA concatemers (contiguous copies of phage genome arising from spontaneously induced lytic cycle phage) would likely pellet with chromosomal DNA and other cellular debris and be removed from exDNA samples. The same linear concatemers, however, would not be excluded in whole-genome preparations, potentially explaining the differences seen in qPCRs of gDNA versus exDNA samples as both single-copy circular and multi-copy concatemer forms of phage DNA contain identical *attP* sites that can be amplified by PCR.

**FIGURE 3:**
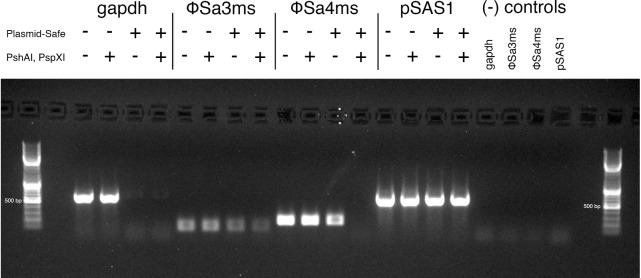
Agarose gel of PCRs from ϕSa4ms selective-depletion experiment. One percent agarose gel stained with SYBR Safe DNA Gel Stain containing end-point PCRs from MSSA476 samples. MSSA476 exDNA was digested with Plasmid-safe exonuclease, *Psh*AI/*Psp*XI restriction endonucleases, both Plasmid-safe and restriction endonuclease, or left untreated with treatment indicated by “+” and “–”. *Gapdh*, ϕSa3ms *attP*, ϕSa4ms *attP*, and pSAS1 target were amplified from treated and untreated samples. ϕSa4ms *attP* target is selectively depleted from combination exonuclease and endonuclease treatment but not solely exonuclease treatment, indicating its existence as a circular element in exDNA samples. Higher levels of ϕSa4ms target compared to ϕSa3ms target correlate with phage enrichment in sequencing. Negative controls for each primer set are shown. The 500 bp ladder band is indicated.

To test this hypothesis, we designed and performed a selective-depletion experiment of excised ϕSa4ms in exDNA samples. Specifically, we examined if ϕSa4ms could be selectively removed from samples only by the combination of ϕSa4ms-specific restriction endonucleases (linearizing circular prophage forms) followed by linear DNA digestion, and not solely by the exonuclease (linear DNase) treatment. ϕSa4ms depletion only by sequential endo- and exonuclease treatment would indicate it exists as a closed circular DNA element. Thus, exDNA samples were treated as described in Section “Materials and Methods” and end-point PCR measurements were performed targeting *gapdh*, the *attP* sites of ϕSa3ms and ϕSa4ms, and the naturally occurring plasmid pSAS1. An agarose gel containing all PCRs is shown in **Figure 3**. *Gapdh* (a marker for linear DNA digestion) was depleted after linear DNase treatment, but remained in high abundance after treatment with the ϕSa4ms-specific restriction endonucleases *Psh*AI and *Psp*XI. ϕSa3ms *attP* target was present in each condition tested, indicating that it can also exist in the circular form in extra-chromosomally enriched DNA samples, however, in lower abundance. The *attP* target of ϕSa4ms is present and in greater abundance than that of ϕSa3ms in untreated, restriction endonuclease-only and linear DNase-only treated samples. Treatment with ϕSa4ms-specific endonuclease followed by linear DNase treatment results in the complete loss of ϕSa4ms target, verifying that ϕSa4ms is indeed abundant relative to ϕSa3ms in extra-chromosomally enriched samples, and that this enrichment is most likely due to circular forms of the prophage element (**Figure 3**). In further support, pSAS1 (a circular plasmid in MSSA476 which does not contain restriction sites for *Psh*AI or *Psp*XI) was not affected by any sample treatment.

### Extra-Chromosomal Localization of ϕSa4ms Circular Prophage Is a Temporal and Likely Rare Event

**FIGURE 4:**
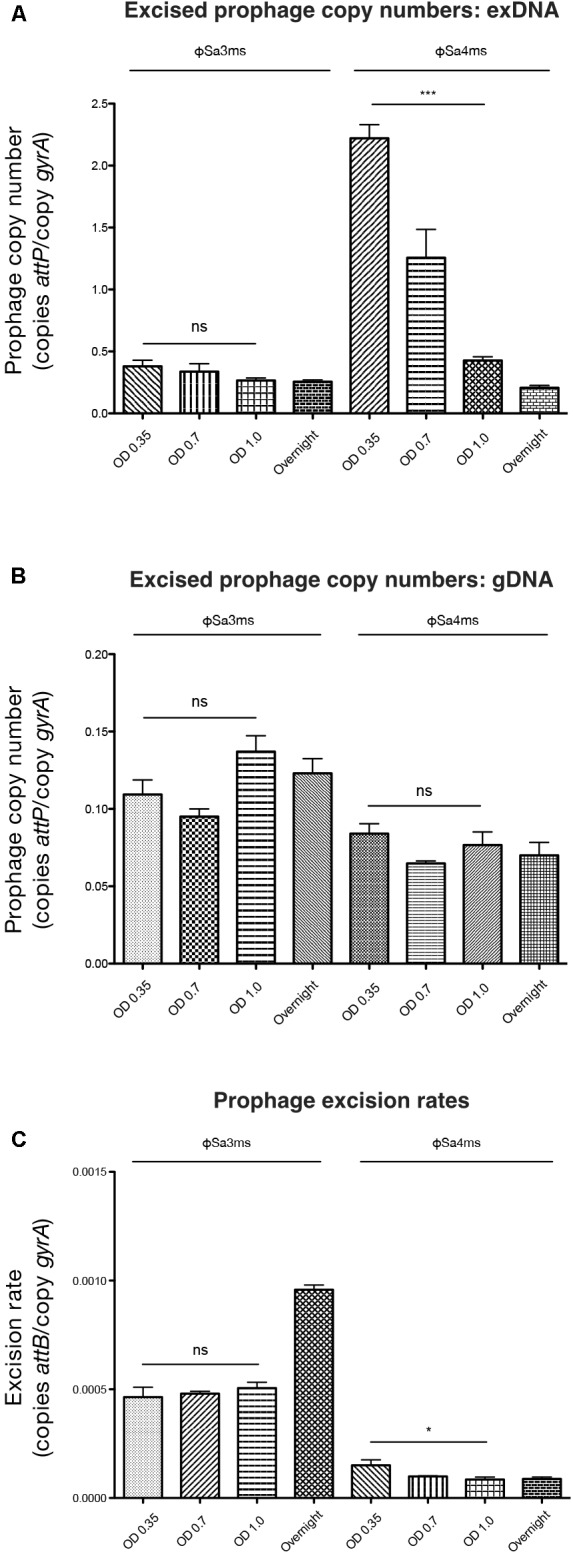
qPCR characterization of MSSA476 prophage excision rates and excised copy numbers from logarithmic and overnight cultures. **(A)** Excised prophage copy numbers (copies *attP*/copy *gyrA*) from MSSA476 extra-chromosomally enriched DNA (exDNA) samples at indicated optical densities. ϕSa3ms does not show a significant difference in excised copy number from OD_600_ = 0.35 to 1.0, whereas ϕSa4ms excised copy number significantly decreases during this interval. **(B)** Prophage copy numbers from MSSA476 whole-genome DNA (gDNA) samples at indicated optical densities. Neither ϕSa3ms nor ϕSa4ms have significantly different excised prophage copy numbers comparing OD_600_ = 0.35 and 1.0 samples. **(C)** Excision rates (copies *attB*/copy *gyrA*) of ϕSa3ms and ϕSa4ms at indicated optical densities. ϕSa3ms does not show a significant difference in excision rate comparing OD_600_ = 0.35 and 1.0 samples, whereas a significant decrease exists for ϕSa4ms. Bars indicate mean ± SEM. ^∗^*P* < 0.05 and ^∗∗∗^*P* < 0.001.

We found episomal, circular copies of ϕSa4ms in the MSSA476 population. However, this enrichment did not appear to correspond to total excised phage copy numbers in PCRs of gDNA. To better understand the episomal nature of ϕSa4ms, we next examined our full qPCR data set (**Table 2**), analyzing changes in ϕSa3ms and ϕSa4ms circular copy numbers (via *attP* in exDNA samples), total excised phage copy numbers (via *attP* in gDNA samples), and excision rates (via *attB* in gDNA samples) over the MSSA476 growth cycle. Examination in this manner allowed insights into MSSA476’s prophage dynamics, and in addition, whether ϕSa4ms may be acting as an active lysogen (i.e., excising into the extra-chromosomal compartment without increases in overall excised copy number). Bar graph visualization of qPCR data from **Table 2** is presented in **Figure 4**.

Analysis of excised prophage copy numbers from exDNA samples revealed that for ϕSa4ms, extra-chromosomal localization of circular prophage is a temporal event, with the highest prevalence of cytoplasmic prophage carriage at early-logarithmic phase. Excised ϕSa4ms copy number in exDNA samples decreased significantly from OD_600_ = 0.35 to 1.0 (*P* < 0.0001), while for ϕSa3ms, there was no significant change in its excised circular copy number over this interval (*P* = 0.0982) (**Table 2** and **Figure 4A**). However, while circular ϕSa4ms significantly decreased in copy number during logarithmic growth, its total excised phage copy number in the MSSA476 population (as measured in gDNA samples, representing circular and concatemer forms) did not change, nor did that of ϕSa3ms. ϕSa3ms and ϕSa4ms did not have significant differences in total excised phage copy numbers from OD_600_ = 0.35 to 1.0 (*P* = 0.118 and *P* = 0.527, respectively) (**Table 2** and **Figure 4B**), indicating that the excision and presence of circular ϕSa4ms does not appear to be a precursor to potential phage linear replication. Interestingly, the excision rate of ϕSa4ms appeared to correlate with its decrease in circular copy number, as it significantly decreased from OD_600_ = 0.35 to 1.0 (*P* = 0.036) (**Table 2** and **Figure 4C**). The ϕSa3ms excision rate did not significantly change during this interval (*P* = 0.2403), however, it did increase approximately twofold from OD_600_ = 0.35 to the O/N time point (**Table 2** and **Figure 4C**).

While these data indicated that ϕSa4ms was temporally localized in the cytoplasm as a circular element as well as potentially an active lysogen in MSSA476 cells, the prevalence of its stable excision was unclear. We employed qPCR targeting pSAS1 (the naturally occurring circular plasmid in MSSA476) as a reference to understand the level of enrichment imparted by our exDNA preparation procedure. We found that pSAS1 existed on average at one to two copies per cell (copies pSAS1 target/copy *gyrA*) in gDNA preparations of MSSA476 at OD_600_ = 0.35 and 0.7, but was enriched ∼1500-fold to an average of 2322 copies pSAS1/copy *gyrA* in exDNA samples (data not shown). That ϕSa4ms copy number in our exDNA qPCR reaches, at a maximum, 2.22 copies *attP*/copy *gyrA* (**Table 2**), suggests that the ϕSa4ms excision we uncover is a rare event. The percentage of cells with stably excised ϕSa4ms, as well as its circular copy number per cell, is presently unclear.

### Promoter Alteration by ϕSa4ms Excision/Integration Affects *htrA_2_* Transcription and Heat-Stress Survival in *S. aureus*

**FIGURE 5:**
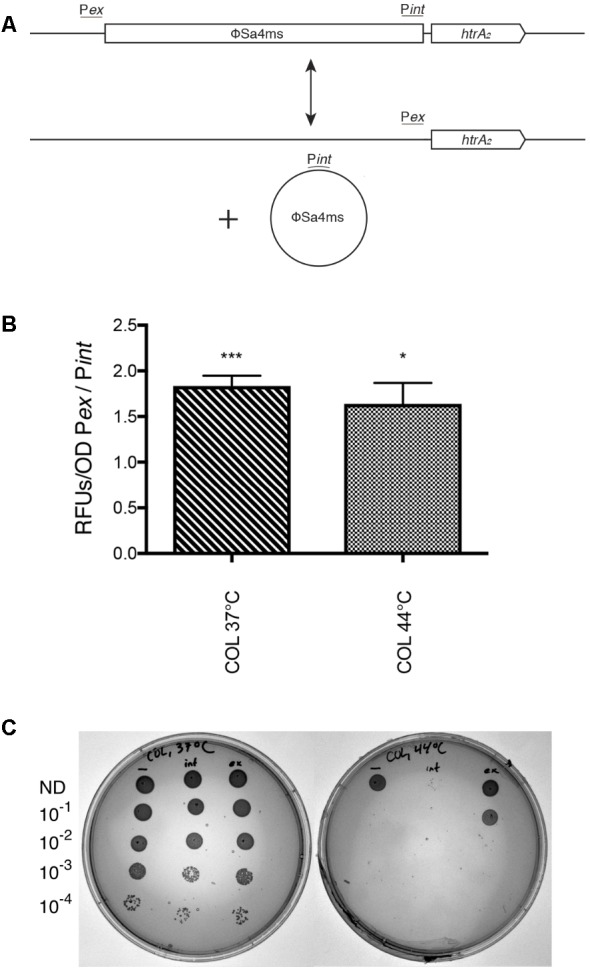
ϕSa4ms excision/integration alters the promoter and transcription of *htrA_2._* **(A)** Diagram illustrating ϕSa4ms integrated and excised states in MSSA476 and the unique DNA regions of the P*int* and P*ex* promoters. *Top*: When ϕSa4ms is integrated in the chromosome, *htrA_2_* is promoted by P*int. Bottom*: ϕSa4ms excision joins the P*ex* region immediately upstream to *htrA_2_*_,_ and the gene is promoted by P*ex*. DNA sequence unique to the P*int* region is carried on circular ϕSa4ms when the phage is excised. **(B)** Ratio of GFP fluorescence from P*ex*- versus P*int*-promoted *gfpmut2* constructs in COL *htrA_2_* deletion strain at 37°C and 44°C. Bars indicate mean ± SEM. Significance as tested by ratio paired *t*-test indicated above each bar. **(C)** Dilution series of COL *htrA_2_* deletion strain complemented with *htrA_2_* promoted by P*int* (int) or P*ex* (ex). The symbol “–” indicates COL *htrA_2_* deletion strain containing empty pCN35 plasmid. Constructs are spotted in a log-dilution series and grown at 37°C (left) or 44°C (right) for 24 h. Dilution factor indicated on left of plates; ND, not diluted. COL constructs display equal survival at 37°C, but COL P*ex* displays 2-log survival above COL P*int* at 44°C. Unexpectedly, uncomplemented COL *htrA_2_* shows 1-log survival above COL P*int*. COL P*int* shows few colonies at 44°C in the undiluted culture spot. ^∗^*P* < 0.05 and ^∗∗∗^*P* < 0.001.

Since qPCR data indicated ϕSa4ms could exist as an active prophage, a stable subpopulation of MSSA476 cells may therefore exist with an altered chromosomal sequence, as ϕSa4ms integrates 30 bp upstream of the stress response serine protease-encoding *htrA_2_*. Sumby and Waldor (2003) previously noted the possibility of altered *htrA_2_* transcription by ϕSa4ms excision/integration, and our data suggested that indeed a subpopulation of MSSA476 cells would harbor *htrA_2_* under an alternative promoter (**Figure 5A**). We therefore developed a P*htrA_2_-gfpmut2* reporter system to examine whether GFP fluorescence would be altered when the GFP-encoding gene *gfpmut2* was under the control of the ϕSa4ms-integrated (P*int*) or ϕSa4ms-excised (P*ex*) promoters. Experiments were performed in a *S. aureus* COL *htrA_2_* deletion knockout, a well-characterized strain where *htrA_2_* was shown to impact heat-stress survival (Rigoulay et al., 2005). Comparisons of GFP fluorescence at 37 and 44°C showed that in COL *htrA_2_*, fluorescence was greater when *gfpmut2* was promoted by P*ex* rather than by P*int*, indicating that P*ex* is a stronger promoter of *htrA_2_* than P*int*. GFP fluorescence was greater in the P*ex* construct at both 37°C (1.83 ± 0.11 P*ex*/P*int* ratio, *P* < 0.001) and 44°C (1.64 ± 0.23 P*ex*/P*int* ratio, *P* = 0.026) (**Figure 5B**).

Due to its reported role in promoting heat-tolerance in COL, we examined if promoter alteration could affect survival of a *htrA_2_*-complemented *S. aureus* COL *htrA_2_* deletion mutant. Complemented strains, containing the full-length *htrA_2_* gene under the control of either the P*int* or P*ex* promoter on the pCN35 plasmid or with empty pCN35 (uncomplemented) were grown in a dilution series at 37 or 44°C on agar plates. COL constructs at 37°C did not display any survival differences among the three strains, however, at 44°C, COL P*ex* displayed approximately 2-log increased survival over COL P*int* (**Figure 5C**, top). Surprisingly, COL P*int* survived 1-log worse than uncomplemented COL *htrA_2_*, indicating that *htrA_2_*-mediated heat-stress survival may depend upon specific promoter sequences and not solely levels of transcription or promoter strength. Environmental conditions may select for cells with one promoter versus another, and ϕSa4ms’s excision/integration dynamics could provide MSSA476 with a potential switching mechanism to create these advantageous subpopulations.

### Episomal Prophages Uncovered in Additional *S. aureus* Clinical Isolates

**FIGURE 6:**
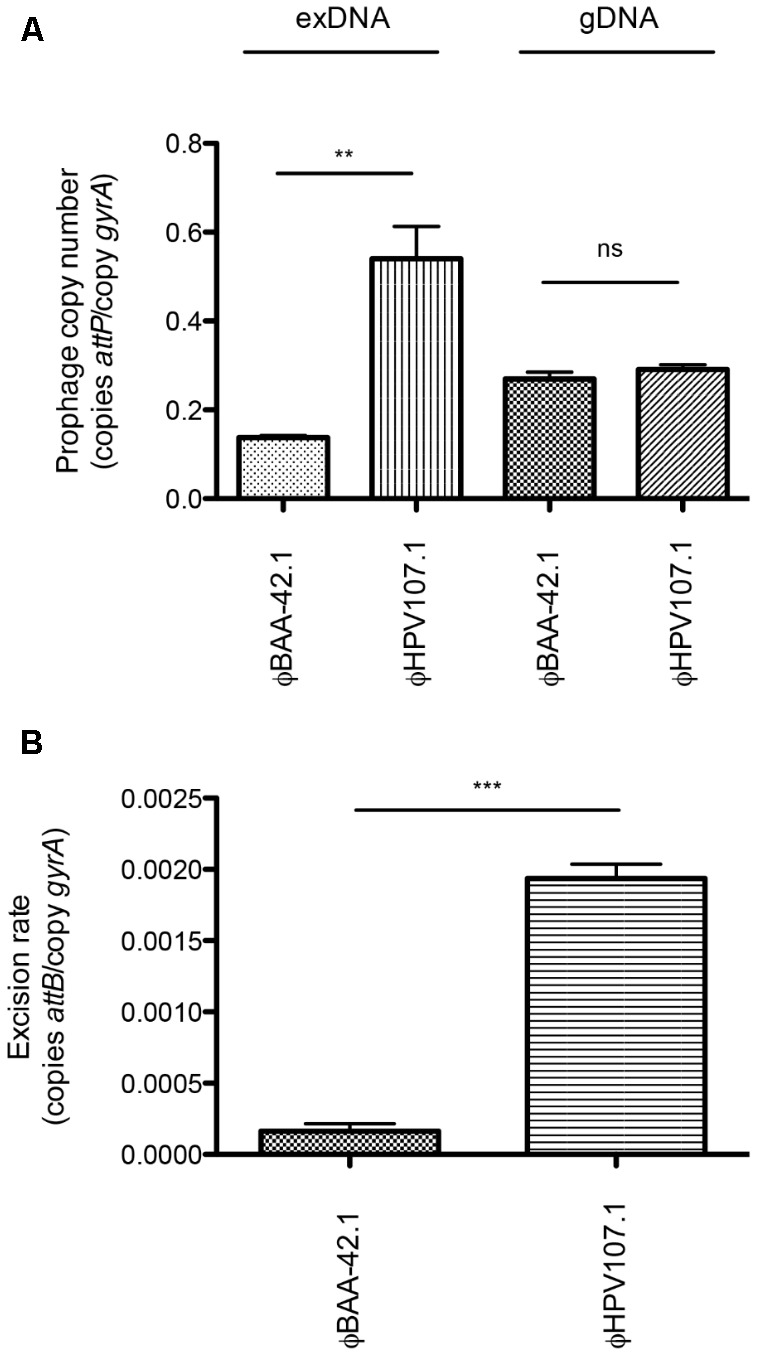
qPCR characterization of *hlb-*converting phages. **(A)** Excised copy numbers (copies *attP*/copy *gyrA*) of *hlb*-converting phages ϕBAA-42.1 and ϕHPV107.1. ϕHPV107.1 has significantly higher copy number than ϕBAA-42.1 in exDNA samples (left), however, gDNA samples (right) reveal no significant difference in excised copy number between ϕBAA-42.1 and ϕHPV107.1. **(B)** Excision rates (copies *attB*/copy *gyrA*) of *hlb*-converting phages ϕBAA-42.1 and ϕHPV107.1. ϕHPV107.1 has a significantly higher excision rate than ϕBAA-42.1. Bars indicate mean ± SEM. ^∗∗^*P* < 0.01 and ^∗∗∗^*P* < 0.001.

In this study, we uncovered and characterized the episomal dynamics of the ϕSa4ms prophage in *S. aureus* MSSA476. Our exDNA sequencing screening, however, also uncovered the presence of other likely episomal prophages in additional clinical isolates (**Table 1**). While we did not characterize the prophage dynamics of these other strains in this study, we were curious if detection of their episomal prophages was also only apparent through exDNA isolation and sequencing, and if their presence would be missed by gDNA-focused approaches. To test this, we performed an inter-strain comparison, examining by qPCR the excision rates and excised prophage copy numbers of *hlb*-converting phages in BAA-42 (containing ϕBAA-42.1) and HPV107 (containing ϕHPV107.1). Sequencing data revealed that only ϕHPV107.1 and not ϕBAA-42.1 was significantly enriched by exDNA isolation (**Table 1**), and qPCR copy number examination of exDNA samples confirmed this result. ϕHPV107.1 had a higher copy number as compared to ϕBAA-42.1 (*P* = 0.0052) in exDNA samples (**Table 3** and **Figure 6A**, left). However, as observed previously with the MSSA476 prophages, copy number data from gDNA samples did not correlate with exDNA sample data. ϕHPV107.1 and ϕBAA-42.1 did not have significant copy number differences in gDNA samples (*P* = 0.3205) (**Table 3** and **Figure 6A**, right), verifying that episomal prophage enrichment is only apparent due to exDNA-focused isolation. Interestingly, when we examined excision rates of the two prophages, we found that in this comparison, ϕHPV107.1 did indeed have the highest excision rate. The excision rate of ϕHPV107.1 was significantly higher than ϕBAA-42.1 (*P* = 0.0009) (**Table 3** and **Figure 6B**). Overall, this result suggests that like ϕSa4ms, ϕHPV107.1 may exist as a circular, episomal DNA element in a higher proportion of the HPV107 population than that of ϕBAA-42.1 in its respective population. The lytic induction of these prophages in other cells of their populations (and the generation of phage genome concatemers), however, may mask uncovering their rarer episomal natures in qPCRs of gDNA samples.

**Table 3:** Excised copy numbers and excision rates of selected *hlb*-converting phages from exDNA sequencing conditions.

Prophage	Excised prophage copy number (copies *attP*/copy *gyrA*)		Excision rate (copies *attB*/copy *gyrA*)
	exDNA	gDNA	
ΦBAA-42.1	0.1380 ± 0.005	0.2696 ± 0.016	1.65 × 10^-4^ ± 5.15 × 10^-5^
ΦHPV107.1	0.5403 ± 0.073	0.2910 ± 0.015	1.94 × 10^-3^ ± 9.94 × 10^-5^

*Values are displayed as mean ± SEM*.

## Discussion

This study explored the prevalence of extra-chromosomal prophages in *S. aureus* as an extension of previous work uncovering the plasmidial phage ϕBU01 in *S. aureus* NRS19 (Utter et al., 2014). We isolated and sequenced the exDNA of 15 clinically relevant *S. aureus* strains with known chromosomal sequences, but unlike our previous study, we did not find any prophages existing as solely plasmidial elements. We did uncover, however, several episomal prophages appearing to exist as circular DNA elements and potential active lysogens that were enriched by our exDNA isolation procedure (**Table 1**). It therefore seems that the existence of plasmidial prophages in *S. aureus* is uncommon and ϕBU01 may represent one of these rare prophages. Episomal prophages, on the other hand, appear to be fairly widespread among *S. aureus* isolates and were identified in 33% of strains (5 of 15) examined in this study.

### Strains and Phages Possess Different Mobilization Capacities With Unclear Mechanisms

We could distinguish strains that carried episomal prophages in the extra-chromosomal compartment from those with no prophages detected, but our study did not reveal why some strains have such a phage-mobilization capacity while others do not. Goerke et al. (2006) demonstrated a similar observation, finding that phages ϕs80b and ϕs84b (*hlb*-converting phages) were integrated within the *hlb* gene in *S. aureus* s64c, but were found to alternate between integrated and extra-chromosomal carriage in strain 8325-4. Both s64c and 8325-4 are phage-cured strains, suggesting that a host-factor (or factors) likely determines phage localization and mobilization capacity within the cell. In a separate manner, phage induction capacity into the lytic cycle has also been observed to depend upon host background, with ϕSa2mw induced by mitomycin C from *S. aureus* strains MW2 and Newman, but not 8325-4, RN6390, or ISP479c (Wirtz et al., 2009). Our data indicate that the phages we describe here are localized and harbored within the extra-chromosomal compartment in a manner distinct from lytic excision and replication (Utter et al., 2014; Deutsch et al., 2016). However, it is possible similar host factors may govern both events. The *hlb*-converting phages we characterized from strains BAA-42 and HPV107 contained high sequence homology over their integration/excision modules; however, they displayed different levels of enrichment in extra-chromosomal sequencing and qPCR characterization, with HPV107 carrying a greater proportion of its *hlb*-converting phage extra-chromosomally. Host differences similar to those affecting ϕs80b and ϕs84b localization may account for the differences in *hlb*-converting phage localization among our strains, although such factors have yet to be uncovered.

Within individual strains, we also noted differences in phage localization. In MSSA476, ϕSa4ms—but not ϕSa3ms—was enriched in sequencing, with the localization of circular, episomal ϕSa4ms confirmed by qPCR and selective-depletion experiments. ϕSa3ms and ϕSa4ms share the same host background, suggesting that a phage factor may be responsible for localization differences, but it is currently unclear which factor(s) could be responsible. Prophage excision requires recombination mediated by integrase; stochastic expression differences of a phage’s integrase or excision-related genes may control localization into the extra-chromosomal compartment. If excision occurs without inducing conditions, then phages could be localized in the cytoplasm in a manner consistent with the lysogenic cycle. Feiner et al. (2015) reviewed such phage activity in a range of bacterial species, particularly those whose excision/integration dynamics can act as a molecular switch for the cell, terming the process “active lysogeny.”

### Episomal Phages Are Only Detected in exDNA and Not gDNA Samples

qPCR characterization of prophage excision rates and excised copy numbers revealed, surprisingly, that episomal prophages are detected only in exDNA samples and that their presence is masked in gDNA preparations. In MSSA476, for example, the overall excised phage copy number as measured from qPCRs of gDNA is relatively low, with ϕSa3ms and ϕSa4ms at 0.095 and 0.0647 copies *attP*/copy *gyrA*, respectively. This would correspond to very low increases in read depth over the ϕSa3ms and ϕSa4ms prophage chromosomal locations if only MSSA476 gDNA was sequenced and read-mapped to a MSSA476 reference chromosome (likely 9.5 and 6.5% maximum increases, respectively). Using gDNA, coverage analysis tools would likely not find regions of significantly higher coverage spanning either prophage location, so while ϕSa3ms copy number was greater than ϕSa4ms in gDNA samples, neither phage would be found as enriched by this approach, and the episomal nature of ϕSa4ms would be overlooked. ϕSa4ms excised phage copy number in exDNA samples, however, is much greater (1.26 copies *attP*/copy *gyrA* at OD_600_ ∼0.7), owing to concurrent enrichment of circular ϕSa4ms and removal of chromosomal DNA in the exDNA isolation procedure, increasing the *attP*/*gyrA* ratio. This increase is sufficient for significantly higher read depth across the prophage location when mapping exDNA sequencing reads to the chromosome. The excised copy number of ϕSa3ms in exDNA samples, while elevated from that seen in gDNA samples (0.34 versus 0.095 copies *attP*/copy *gyrA*, respectively), was not high enough to have significantly higher read depth or coverage over the ϕSa3ms genome location in MSSA476 read-mappings. Thus, our exDNA isolation and sequencing approach allows the distinction of episomal elements, even when such elements would be masked in qPCR and sequencing of DNA prepared by other methods.

**FIGURE 7:**
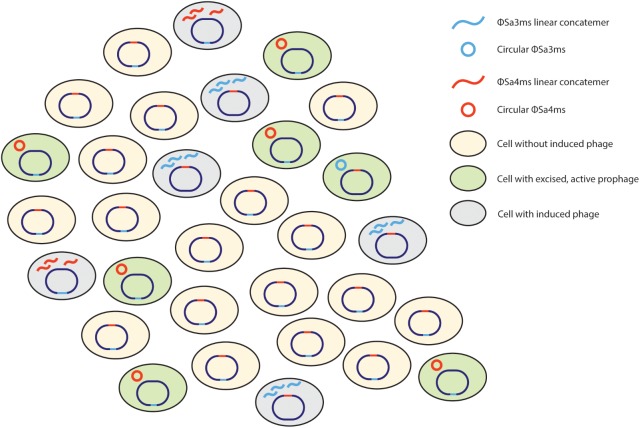
Qualitative model of MSSA476 early-logarithmic culture. At early logarithmic phase, the MSSA476 population contains cells with phages in lytic, lysogenic, and active lysogenic cycles. The vast majority of cells are stably lysogenized (tan) with integrated ϕSa3ms (blue) and ϕSa4ms (orange). Subpopulations of cells contain excised prophage elements that appear to be in an active lysogenic cycle (green). Of these cells, those with ϕSa4ms excised outnumber those with ϕSa3ms excised. Subpopulations of cells with phage induced into the lytic cycle are pictured in gray. Here, ϕSa3ms-induced cells outnumber ϕSa4ms-induced cells. Subpopulations are shown at relative percentages for illustrative purposes, rather than quantitative levels suggested by qPCR and other data.

Additionally, we showed that our exDNA isolation procedure enriches circular DNA elements, allowing the identification of episomal and plasmidal elements, and that whole-genome focused approaches do not impart such selectivity in sample preparations. Higher excision rates and higher total excised phage copy numbers (from gDNA samples) did not correlate with episomal prophage enrichment in our NGS screening. We hypothesize that the reason ϕSa4ms copy number was lower than that of ϕSa3ms in gDNA samples, despite the presence of ϕSa4ms episomes, is likely due to the presence of more linear, lytic-cycle phage genome concatemers of ϕSa3ms in the MSSA476 population. The greater number of linear phage concatemers in gDNA samples (but not in exDNA) likely masks the low percentage of circular phage elements, preventing their discovery in sequencing or qPCR in the absence of exDNA enrichment. This is supported by the fact that the excised prophage copy numbers of ϕSa3ms and ϕSa4ms are still very low in exDNA samples despite the potential ∼1500-fold enrichment of circular DNA (i.e., pSAS1 plasmid) by our protocol. Further, this suggests that for MSSA476, both ϕSa3ms and ϕSa4ms excised copy numbers are primarily composed of linear elements, which are likely from a small subset of cells whose phages are undergoing spontaneous lytic cycle replication. While both phages in MSSA476 are capable of such lytic events, only ϕSa4ms showed enrichment for circular prophage elements in its cytoplasm. This result might indicate that for a number of cells in the MSSA476 population, circular ϕSa4ms prophage is not participating in the lytic cycle, but is perhaps in a state akin to active lysogeny (Feiner et al., 2015). Other individual cells in the MSSA476 population, however, appear to contain ϕSa3ms or ϕSa4ms undergoing lytic cycle replication. A model for how these bacteriophages might exist in the MSSA476 population during early-logarithmic phase is presented in **Figure 7**.

### ExDNA Isolation and Sequencing Reveals Potential and Previously Identified Active Prophages

Having better understood the nature of enrichment imparted by exDNA isolation (i.e., enrichment of circular DNA elements), we were able to analyze our full MSSA476 qPCR dataset (**Table 2**) and uncover the strain’s prophage dynamics. Surprisingly, results revealed that excision of circular ϕSa4ms into the extra-chromosomal compartment was temporal, with the highest levels of circular prophage recorded in early-logarithmic growth, and that its circular copy number rapidly decreased with increasing cell-density. Furthermore, these decreases occurred without any concurrent increase in overall ϕSa4ms copy number (as measured by gDNA PCRs), indicating that ϕSa4ms circular elements were not precursors to production of linear phage genome concatemers and that the prophage was likely not participating in the lytic cycle. In addition, these data show that phage enrichment would likely go undetected in MSSA476 if samples were prepared at OD_600_ ≥ 1.0. Therefore, it may be beneficial to purify exDNA at multiple time points in future studies, elucidating the entire mobilization dynamics of a bacterial genome over its growth cycle.

The time-course qPCR data also showed that the excision rate and circular copy number of ϕSa4ms decreased from early to later-log phase, suggesting potential control of this event by the bacterial cell, with some factors either promoting excision at early-logarithmic growth, or perhaps increased re-integration at later growth points. In addition, whether ϕSa4ms or related circular prophage elements undergo any replication or remain as single copy in this excised state is unclear. Goerke et al. (2006) reported Hlb^-^/sak^2^ *S. aureus* strains with multiple *hlb*-converting phages integrated in the chromosome. Replication of excised prophage could be the first step in generating such strains. Our qPCR approach examined bacterial populations as a whole, but cannot address these specific questions; further experiments on the single-cell level are necessary to uncover such exact excision/integration (and potentially replication) dynamics.

The distinction between phages in the lytic versus lysogenic cycle is important when considering the roles of prophages as molecular switches (i.e., active lysogens) or as mechanisms to generate diversity in bacterial populations. Excision by phage induction into the lytic cycle likely leads to cell death, whereas excision in active lysogeny generates stable diversity in the population that can be passed to daughter cells. We found that ϕSa4ms excision/integration alters the promoter sequence and consequent transcription of *htrA_2_* in a GFP-reporter system and affects survival of *S. aureus* COL to heat-stress. While qPCR revealed that only a minor proportion of MSSA476 likely carries excised ϕSa4ms and alternatively promoted *htrA_2_*, heat-stress or other environmental factors could select for and expand this subpopulation. Our work suggests the behavior of ϕSa4ms could generate a small subset of cells in the MSSA476 population better equipped to respond to and survive environmental stress. Studying strains by our extra-chromosomal sequencing approach may be useful for researchers seeking active prophages or similar-acting DNA elements in other species of interest. As further proof of this concept, we performed a “retroactive” exDNA enrichment and sequencing of *S. pyogenes* SF370, which indeed showed the expected enrichment of sequences for the previously reported episomal phage-like element SpyCIM1, which acts as a switch in the strain’s mismatch repair operon (Scott et al., 2008; Deutsch et al., 2016). Indeed, a similar exDNA enrichment and sequencing approach was used to identify mobile retrotransposons in Arabidopsis and rice (Lanciano et al., 2017).

In addition to ϕSa4ms, the intergenic prophages we uncovered as enriched in our *S. aureus* screening included one prophage in HPV107 (unclear integrase type, intergenic between tRNA-Ser and enterotoxin), and two prophages in NRS22 and BK2529 [both with Sa7-like integrases, and intergenic between *rpmF* (50S ribosomal protein L32) and *isdB* (iron-regulated surface determinant)]. The potential effects of their extra-chromosomal carriage on hosts are unclear, but phage integration in these loci has been reported previously (Bae et al., 2006; Goerke et al., 2006). The typically intragenic prophages we found enriched in our screening included a *geh*-converting prophage in *S. aureus* NRS143, two prophages in HPV107 (an *hlb*-converting phage, and a 6-phospho-β-galactosidase-integrating phage), and two prophages in NRS22 (one prophage integrated within a hypothetical protein-encoding gene, and one prophage disrupting a radical SAM-encoding gene). In addition, we uncovered an enriched ICE*6013*-like element, whose localization has been previously reported as both integrated and extra-chromosomal (Smyth and Robinson, 2009; Sansevere et al., 2017). The *hlb*- and *geh*-converting phages could potentially act as switches to control the expression of these virulence factors and future work should investigate this possibility. Likewise, the effects of the other intragenic prophages are unclear, however, their enrichment suggests that they could also act as switches to control underlying gene expression. Disruption of genes by the intragenic prophages in NRS22 has been reported previously in other *S. aureus* strains (Bae et al., 2006). However, to our knowledge, disruption of a 6-phospho-β-galactosidase encoding gene (HPV107) has not been reported previously and represents a novel prophage integration site. The role of the ICE is also unclear, but its potential as a switch is intriguing.

Overall, exDNA isolation and sequencing revealed the episomal nature of specific typically integrated staphylococcal prophages. Previously, the movement of *hlb*-converting phages to atypical chromosomal loci was shown to occur in isolates from cystic fibrosis and bacteremic patients with phage mobilization thought to be important for invasive infection. As also noted by (Goerke et al., 2006), the localization of typically integrated prophages into the cytoplasm (without lytic induction) could precede generation of such unique CF and bacteremic *S. aureus* isolates. Our extra-chromosomal enrichment and sequencing approach may allow characterization of strains for such a mobilization capacity or potential. In addition, uncovering the episomal nature of other prophages (e.g., *geh*-converting, 6-phospho-β-galactosidase-disrupting prophages) may help better direct future research efforts and allow discovery of novel processes and events important to *S. aureus* infection.

## Conclusion

This study screened the exDNA of 15 clinical *S. aureus* isolates, uncovering the prevalence of episomal prophages with potential roles in virulence factor expression and regulation. MSSA476 qPCR characterization revealed that prophage ϕSa4ms can exist as an episomal circular DNA element that does not appear to be a precursor to lytic cycle replication. Importantly, we found that episomal prophages are only detectable in extra-chromosomally enriched DNA samples, and that their presence would have been missed in sequencing or qPCRs of whole-genome DNA samples. Lastly, we showed that ϕSa4ms may act as a potential phage-molecular switch, as its excision/integration alters the promoter sequence of *htrA_2_*, changing its transcription levels and affecting heat-stress survival in *S. aureus* COL. We believe ϕSa4ms behavior represents that of an active lysogen, and that the other episomal prophages we identify may exhibit similar activity. Episomal prophages promote the generation of stable, diverse subpopulations with the potential to impact bacterial infection. exDNA enrichment and sequencing should allow the increased discovery of such elements in *S. aureus* and other bacterial pathogens, and help researchers better assess those genes under control by active lysogeny.

## Author Contributions

DD, BU, and VF conceived and designed the experiments. DD, BU, KV, HS, and LT performed the experiments. DD, BU, KV, HS, LT, and VF contributed materials and analyzed the data. DD and VF wrote the paper.

## Conflict of Interest Statement

The authors declare that the research was conducted in the absence of any commercial or financial relationships that could be construed as a potential conflict of interest. The opinions, interpretations, conclusions, and recommendations contained herein are those of the authors and are not necessarily endorsed by the FDA.

## References

[B1] Bae T. Baba T. Hiramatsu K. Schneewind O. (2006). Prophages of *Staphylococcus aureus* Newman and their contribution to virulence. *Mol. Microbiol.* 62 1035–1047. 10.1111/j.1365-2958.2006.05441.x 17078814

[B2] Beres S. B. Musser J. M. (2007). Contribution of exogenous genetic elements to the group A Streptococcus metagenome. *PLoS One* 2:e800. 10.1371/journal.pone.0000800 17726530 PMC1949102

[B3] Charpentier E. Anton A. I. Barry P. Alfonso B. Fang Y. Novick R. P. (2004). Novel cassette-based shuttle vector system for gram-positive bacteria. *Appl. Environ. Microbiol.* 70 6076–6085. 10.1128/AEM.70.10.6076-6085.2004 15466553 PMC522135

[B4] Coleman D. C. Sullivan D. J. Russell R. J. Arbuthnott J. P. Carey B. F. Pomeroy H. M. (1989). *Staphylococcus aureus* bacteriophages mediating the simultaneous lysogenic conversion of beta-lysin, staphylokinase and enterotoxin A: molecular mechanism of triple conversion. *J. Gen. Microbiol.* 135 1679–1697. 2533245 10.1099/00221287-135-6-1679

[B5] Deutsch D. R. Utter B. Fischetti V. A. (2016). Uncovering novel mobile genetic elements and their dynamics through an extra-chromosomal sequencing approach. *Mob. Genet. Elements* 6:e1189987. 10.1080/2159256X.2016.1189987 27581613 PMC4993567

[B6] Feiner R. Argov T. Rabinovich L. Sigal N. Borovok I. Herskovits A. A. (2015). A new perspective on lysogeny: prophages as active regulatory switches of bacteria. *Nat. Publish. Group* 13 641–650. 10.1038/nrmicro3527 26373372

[B7] Goerke C. Goerke C. Pantucek R. Pantucek R. Holtfreter S. Holtfreter S. (2009). Diversity of prophages in dominant *Staphylococcus aureus* clonal lineages. *J. Bacteriol.* 191 3462–3468. 10.1128/JB.01804-08 19329640 PMC2681900

[B8] Goerke C. Matias y Papenberg S. Dasbach S. Dietz K. Ziebach R. Kahl B. C. (2004). Increased frequency of genomic alterations in *Staphylococcus aureus* during chronic infection is in part due to phage mobilization. *J. Infect. Dis.* 189 724–734. 10.1086/381502 14767828

[B9] Goerke C. Wirtz C. Wirtz C. Flückiger U. Flückiger U. Wolz C. (2006). Extensive phage dynamics in *Staphylococcus aureus* contributes to adaptation to the human host during infection. *Mol. Microbiol.* 61 1673–1685. 10.1111/j.1365-2958.2006.05354.x 16968231

[B10] Heler R. Samai P. Modell J. W. Weiner C. Goldberg G. W. Bikard D. (2015). Cas9 specifies functional viral targets during CRISPR-Cas adaptation. *Nature* 519 199–202. 10.1038/nature14245 25707807 PMC4385744

[B11] Hendrickson C. Euler C. W. Nguyen S. V. Rahman M. McCullor K. A. King C. J. (2015). Elimination of Chromosomal Island SpyCIM1 from *Streptococcus pyogenes* strain SF370 reverses the mutator phenotype and alters global transcription. *PLoS One* 10:e0145884. 10.1371/journal.pone.0145884 26701803 PMC4689407

[B12] Holden M. T. Feil E. J. Lindsay J. A. Peacock S. J. Day N. P. Enright M. C. (2004). Complete genomes of two clinical *Staphylococcus aureus* strains: evidence for the rapid evolution of virulence and drug resistance. *Proc. Natl. Acad. Sci. U.S.A.* 101 9786–9791. 10.1073/pnas.0402521101 15213324 PMC470752

[B13] Hu C. Hu C. Xiong N. Xiong N. Zhang Y. Zhang Y. (2012). Functional characterization of lipase in the pathogenesis of *Staphylococcus aureus*. *Biochem. Biophys. Res. Commun.* 419 617–620. 10.1016/j.bbrc.2012.02.057 22369949

[B14] Kaneko J. Kimura T. Narita S. Tomita T. Kamio Y. (1998). Complete nucleotide sequence and molecular characterization of the temperate staphylococcal bacteriophage phiPVL carrying Panton-Valentine leukocidin genes. *Gene* 215 57–67. 9666077 10.1016/s0378-1119(98)00278-9

[B15] Katayama Y. Baba T. Sekine M. Fukuda M. Hiramatsu K. (2013). Beta-hemolysin promotes skin colonization by *Staphylococcus aureus*. *J. Bacteriol.* 195 1194–1203. 10.1128/JB.01786-12 23292775 PMC3592002

[B16] Kreiswirth B. N. Löfdahl S. Betley M. J. O’Reilly M. Schlievert P. M. Bergdoll M. S. (1983). The toxic shock syndrome exotoxin structural gene is not detectably transmitted by a prophage. *Nature* 305 709–712. 6226876 10.1038/305709a0

[B17] Kunkel B. Losick R. Stragier P. (1990). The *Bacillus subtilis* gene for the development transcription factor sigma K is generated by excision of a dispensable DNA element containing a sporulation recombinase gene. *Genes Dev.* 4 525–535. 2163341 10.1101/gad.4.4.525

[B18] Lanciano S. Carpentier M.-C. Llauro C. Jobet E. Robakowska-Hyzorek D. Lasserre E. (2017). Sequencing the extrachromosomal circular mobilome reveals retrotransposon activity in plants. *PLoS Genet.* 13:e1006630. 10.1371/journal.pgen.1006630 28212378 PMC5338827

[B19] Lee C. Y. Iandolo J. J. (1986). Lysogenic conversion of staphylococcal lipase is caused by insertion of the bacteriophage L54a genome into the lipase structural gene. *J. Bacteriol.* 166 385–391. 3009394 10.1128/jb.166.2.385-391.1986 PMC214616

[B20] Olson M. E. (2016). Bacteriophage transduction in *Staphylococcus aureus*. *Methods Mol. Biol.* 1373 69–74. 10.1007/7651_2014_186 25646608

[B21] Qu T. Feng Y. Jiang Y. Zhu P. Wei Z. Chen Y. (2014). Whole genome analysis of a community-associated methicillin-resistant *Staphylococcus aureus* ST59 isolate from a case of human sepsis and severe pneumonia in China. *PLoS One* 9:e89235. 10.1371/journal.pone.0089235 24586619 PMC3930696

[B22] Rabinovich L. Sigal N. Borovok I. Nir-Paz R. Herskovits A. A. (2012). Prophage excision activates Listeria competence genes that promote phagosomal escape and virulence. *Cell* 150 792–802. 10.1016/j.cell.2012.06.036 22901809

[B23] Rigoulay C. Entenza J. M. Halpern D. Widmer E. Moreillon P. Poquet I. (2005). Comparative analysis of the roles of HtrA-like surface proteases in two virulent *Staphylococcus aureus* strains. *Infect. Immun.* 73 563–572. 10.1128/IAI.73.1.563-572.2005 15618196 PMC538960

[B24] Sansevere E. A. Luo X. Park J. Y. Yoon S. Seo K. S. Robinson D. A. (2017). Transposase-mediated excision, conjugative transfer, and diversity of ICE6013 elements in *Staphylococcus aureus*. *J. Bacteriol.* 199 e629–e616. 10.1128/JB.00629-16 28138100 PMC5370420

[B25] Scott J. Thompson-Mayberry P. Lahmamsi S. King C. J. McShan W. M. (2008). Phage-associated mutator phenotype in Group A Streptococcus. *J. Bacteriol.* 190 6290–6301. 10.1128/JB.01569-07 18676670 PMC2565987

[B26] Smyth D. S. Robinson D. A. (2009). Integrative and sequence characteristics of a novel genetic element, ICE6013, in *Staphylococcus aureus*. *J. Bacteriol.* 191 5964–5975. 10.1128/JB.00352-09 19648240 PMC2747909

[B27] Sumby P. Waldor M. K. (2003). Transcription of the toxin genes present within the Staphylococcal phage phiSa3ms is intimately linked with the phage’s life cycle. *J. Bacteriol.* 185 6841–6851. 14617648 10.1128/JB.185.23.6841-6851.2003 PMC262704

[B28] Takemaru K. Mizuno M. Sato T. Takeuchi M. Kobayashi Y. (1995). Complete nucleotide sequence of a skin element excised by DNA rearrangement during sporulation in *Bacillus subtilis*. *Microbiology* 141(Pt 2), 323–327. 10.1099/13500872-141-2-323 7704261

[B29] Thammavongsa V. Kim H. K. Missiakas D. Schneewind O. (2015). Staphylococcal manipulation of host immune responses. *Nat. Rev. Microbiol.* 13 529–543. 10.1038/nrmicro3521 26272408 PMC4625792

[B30] Thomer L. Schneewind O. Missiakas D. (2016). Pathogenesis of *Staphylococcus aureus* bloodstream infections. *Annu. Rev. Pathol.* 11 343–364. 10.1146/annurev-pathol-012615-044351 26925499 PMC5068359

[B31] Utter B. Deutsch D. R. Schuch R. Winer B. Y. Verratti K. Bishop-Lilly K. (2014). Beyond the chromosome: the prevalence of unique extra-chromosomal bacteriophages with integrated virulence genes in pathogenic *Staphylococcus aureus*. *PLoS One* 9:e100502. 10.1371/journal.pone.0100502 24963913 PMC4070920

[B32] van Wamel W. J. Rooijakkers S. H. Ruyken M. van Kessel K. P. van Strijp J. A. (2006). The innate immune modulators staphylococcal complement inhibitor and chemotaxis inhibitory protein of *Staphylococcus aureus* are located on beta-hemolysin-converting bacteriophages. *J. Bacteriol.* 188 1310–1315. 10.1128/JB.188.4.1310-1315.2006 16452413 PMC1367213

[B33] Wirtz C. Witte W. Wolz C. Goerke C. (2009). Transcription of the phage-encoded Panton-Valentine leukocidin of *Staphylococcus aureus* is dependent on the phage life-cycle and on the host background. *Microbiology* 155 3491–3499. 10.1099/mic.0.032466-0 19661179

[B34] Yamaguchi T. Hayashi T. Takami H. Nakasone K. Ohnishi M. Nakayama K. (2000). Phage conversion of exfoliative toxin A production in *Staphylococcus aureus*. *Mol. Microbiol.* 38 694–705. 11115106 10.1046/j.1365-2958.2000.02169.x

