# Supplementary material for: Extra-Chromosomal DNA Sequencing Reveals Episomal Prophages Capable of Impacting Virulence Factor Expression in Staphylococcus aureus
Source: Front Microbiol. 2018 Jul 2;9:1406. doi: 10.3389/fmicb.2018.01406 (PMC6036120; doi:10.3389/fmicb.2018.01406)
Supplement: Supplementary file 1 [file Table_1.docx]

Supplementary Table 1

Extra-Chromosomal DNA Sequencing Reveals Episomal Prophages Capable of Impacting Virulence Factor Expression in *Staphylococcus aureus*

**Douglas R. Deutsch, Bryan Utter, Kathleen J. Verratti, Heike Sichtig, Luke J. Tallon, Vincent A. Fischetti^*^**

*** Correspondence:** Dr. Vincent A. Fischetti, vaf@rockefeller.edu

**Table S1. Strains and constructs used in this study.**

| **Strains** | **Notes** | **Source** |  |
| --- | --- | --- | --- |
|  |  |  |  |
|  |  |  |  |
| ***E. coli*** |  |  |  |
|  |  |  |  |
| *E. coli* TOP10 | Cloning host | Invitrogen |  |
| *E. coli* DC10B | Cloning host | Lab strain |  |
| *E. coli* DH5⍺::pCN35 | Strain containing pCN35 plasmid | (Charpentier et al., 2004) |  |
| *E. coli* DH5⍺::pCN56 | Straining containing pCN56 plasmid | (Charpentier et al., 2004) |  |
|  |  |  |  |
|  |  |  |  |
| ***S. aureus*** |  |  |  |
|  |  |  |  |
|  |  |  |  |
| *RN4220* | Restriction deficient cloning host | (Kreiswirth et al., 1983) |  |
| COL *htrA2* | COL, *htrA_2_*::*spc* Spc^R^ | (Rigoulay et al., 2005) |  |
| COL *htrA2* DD11 | COL, *htrA_2_*::*spc* Spc^R^, harboring pCN35, Erm^R^ | This work |  |
| COL *htrA2* DD12 | COL, *htrA_2_*::*spc* Spc^R^, harboring pCN35 with full length *htrA_2_* and 250 bp *int*-promoter, Erm^R^ | This work |  |
| COL *htrA2* DD13 | COL, *htrA_2_*::*spc* Spc^R^, harboring pCN35 with full length *htrA_2_* and 250 bp *ex*-promoter, Erm^R^ | This work |  |
| COL *htrA2* DD14 | COL, *htrA_2_*::*spc* Spc^R^, harboring pCN56, Erm^R^ | This work |  |
| COL *htrA2* DD15 | COL, *htrA_2_*::*spc* Spc^R^, harboring pCN56 with P*htrA_2_*-*int* insert, Erm^R^ | This work |  |
| COL *htrA2* DD16 | COL, *htrA_2_*::*spc* Spc^R^, harboring pCN56 with P*htrA_2_*-*ex* insert, Erm^R^ | This work |  |
|  |  |  |  |
|  |  |  | **Genome Accessions for read-mapping** |
|  |  |  |  |
| BAA-42 | MRSA | ATCC | JXZF01000000 |
| BK2529 | 1996; USA; SCCmecIV, MRSA | VAF | JYBA01000000 |
| E2125 | MRSA, Denmark, SCCmec1, 1964 | VAF | JYAX01000000 |
| HDE288 | 1996; Portugal, SCCmecIVvar; MRSA | VAF | JYAZ01000000 |
| HPV107 | 1992; MRSA; Portugal; SCCmec1a, | VAF | JYAY01000000 |
| MSSA476 | MSSA, Hyper-virulent, community acquired | ATCC | JXZG01000000 |
| NRS2 | Mu3; ATCC 700698 ; Purulent sputum, cardio thoracic surgery, Japan, Oxacillin and Tet resistant; VISA= MIC=4. MRSA; white colonies, robust growth on BHI | NARSA | JYAO01000000 |
| NRS22 | VISA, MRSA inpatient ICU, bloodstream | NARSA | JYAG01000000 |
| NRS127 | MRSA sputum source from Tenn, oxacillin, penicillin, cipro and erythromycin resistant | NARSA | JXZZ01000000 |
| NRS143 | MSSA | NARSA | JYAH01000000 |
| NRS153 | MRSA | NARSA | JXZY01000000 |
| NRS156 | MSSA, vaginal tampon isolate | NARSA | JXZU01000000 |
| NRS158 | MSSA HT 2000 0319, Strain was isolated from skin infection in France | NARSA | JYAN01000000 |
| NRS271 | MRSA, Linezolid-resistant, wound isolate | NARSA | JXZW01000000 |
| NRS387 | MRSA, wound Washington State | NARSA | JYAD01000000 |

| ATCC = American Type Culture Collection |
| --- |
| NARSA = Network on Antimicrobial Resistance in Staphylococcus Aureus  VAF = Vincent A. Fischetti |
