# Supplementary material for: Extra-Chromosomal DNA Sequencing Reveals Episomal Prophages Capable of Impacting Virulence Factor Expression in Staphylococcus aureus
Source: Front Microbiol. 2018 Jul 2;9:1406. doi: 10.3389/fmicb.2018.01406 (PMC6036120; doi:10.3389/fmicb.2018.01406)
Supplement: Supplementary file 2 [file Table_2.docx]

Supplementary Table 2

Extra-Chromosomal DNA Sequencing Reveals Episomal Prophages Capable of Impacting Virulence Factor Expression in *Staphylococcus aureus*

**Douglas R. Deutsch, Bryan Utter, Kathleen J. Verratti, Heike Sichtig, Luke J. Tallon, Vincent A. Fischetti^*^**

*** Correspondence:** Dr. Vincent A. Fischetti, vaf@rockefeller.edu

**Table S2. Primers and probes used in this study.**

| **Primers** | **Sequence** | **Notes** |
| --- | --- | --- |
|  |  |  |
| **End-point PCR/qPCR primers** |  |  |
|  |  |  |
| gyrA_F | CGTGAAGGTGACGAAGTTGT | For per cell normalization |
| gyrA_probe | TGTTTGCATGAGCTACATCAAGCCC | 5' 6-FAM/ZEN/3' IBFQ |
| gyrA_R | CCTTTACCACCACGATTTGA | For per cell normalization |
|  |  |  |
| phihlb_attB_F | ACGTTTATATGTTATCGACCGT | for excision rates of hlb-converting phage |
| phihlb_attB_probe | ACGCGCTGATTTAATCGGACAATCTTCT | 5' 6-FAM/ZEN/3' IBFQ |
| phihlb_attB_R | TTGTCTGATGCACCATTATCA | for excision rates of hlb-converting phage |
|  |  |  |
| phiSa4ms_attB_F | GACGCTTACGTCGGTACT | for φSa4ms excision rates |
| phiSa4ms_attB_probe | ACCAATATCCACTAATGTCCACTCCATTCA | 5' 6-FAM/ZEN/3' IBFQ |
| phiSa4ms_attB_R | TTGATGTGAAGCGGACAATC | for φSa4ms excision rates |
|  |  |  |
| hlb_attP_F | AAAGTCTCCAGTTTGGATACATAGA | for hlb-converting phage copy number and φSa3ms target |
| hlb_attP_probe | CAACAGTATTTATTGGGTTTGGAGTCC | 5' 6-FAM/ZEN/3' IBFQ |
| hlb_attP_R | GAAAGTATGTAATTTAGGGACCCATTAG | for hlb-converting phage copy number and φSa3ms target |
|  |  |  |
| phiSa4ms_attP_F | CCTTGCAACACATTCTGAACAC | for φSa3ms copy number and φSa4ms target |
| phiSa4ms_attP_probe | ACGGCCATTCTCAAACGTACACGA | 5' 6-FAM/ZEN/3' IBFQ |
| phiSa4ms_attP_R | TAAGAGCAAACACGAGTGGAAA | for φSa3ms copy number and φSa4ms target |
|  |  |  |
| pSAS1_F | CCTCGGAACCCTTAACAATCC | for pSAS1 copy number |
| pSAS1_probe | ATGGTCGGCTTAATAGCTCACGCT | 5' 6-FAM/ZEN/3' IBFQ |
| pSAS1_R | GCGTTGAGAAGAACCCTTAACTA | for pSAS1 copy number |
|  |  |  |
| gapdh_PCR_F | GTCAACGAATATTGCAATTAATGGTATGG | for measuring *gapdh* target |
| gapdh_PCR_R | CGCACCAGTAGAAGTAGGAATAATGC | for measuring *gapdh* target |
|  |  |  |
| pSAS1_PCR_F | GAAGGTCGTCTATCTCTCAGATGTC | for measuring pSAS1 target (end-point PCR only) |
| pSAS1_PCR_R | AAGGATGGTCTCAAGAGGAATTAGCC | for measuring pSAS1 target (end-point PCR only) |
|  |  |  |
|  |  |  |
| **P*int*/*ex* and *htrA_2_*- related primers** |  |  |
|  |  |  |
| Pint_upstm | GCTTATGGATCCATAAATGATCAAACCACACCACCT | 250bp upstream of *htrA_2_* gene, phiSa4ms integrated primer, BamHI restriction site, for P*int* construciton |
| Pex_upstm | GCTTATGGATCCAAAAATCGCATAAATAATTGATGTGAAG | 250bp upstream of *htrA_2_* gene, phiSa4ms excised primer, BamHI restriction site, for P*ex* construction |
| P_dwnstm | GCTTATGGTACCCTAAGGAATTACATGTTTTTTACCAATATC | 30bp within 5’ end of *htrA_2_* gene, Kpn1 restriction site, for P*int*/*ex* construction |
|  |  |  |
| Pint_upstm_seq | ATAAATGATCAAACCACACCACCT | for Sanger sequencing of P*int* |
| Pex_upstm_seq | AAAAATCGCATAAATAATTGATGTGAAG | for Sanger sequencing of P*ex* |
| P_dwnstm_seq | CCTAAGGAATTACATGTTTTTTACCAATATC | for Sanger sequencing of P*int*/P*ex* constructs |
|  |  |  |
| full_htrA2_dwnstm | GCTTATGGTACCTTATTTTAGTTTAATATTAATTTCTTTC | 3' end of *htrA_2_*, for complementation of *htrA_2_*, Kpn1 restriction site |
| full_htrA2_seq1 | GTACTGACTTTTAGGAATTACATGT | For Sanger sequencing of *htrA_2_* constructs |
| full_htrA2_seq2 | GTAATTACTGAATTAGATGGC | For Sanger sequencing of *htrA_2_* constructs |
| full_htrA2_seq3 | GTCTGAAACGGTGGGATATC | For Sanger sequencing of *htrA_2_* constructs |
